# Soybean Soluble Polysaccharides: Composition, Structure, and Protein Stabilization Mechanism in Acidic Milk Drinks

**DOI:** 10.3390/foods14213629

**Published:** 2025-10-24

**Authors:** Yujian Li, Guijiang Liang, Zhaojun Wang, Maomao Zeng, Zhiyong He, Qiuming Chen, Fang Qin, Jie Chen

**Affiliations:** 1 State Key Laboratory of Food Science and Resources, Jiangnan University, Wuxi 214122, China; lyjdgzyx@163.com (Y.L.); liangguijiang0103@163.com (G.L.); zhaojun.wang@jiangnan.edu.cn (Z.W.); mmzeng@jiangnan.edu.cn (M.Z.); zyhe@jiangnan.edu.cn (Z.H.); chenqm@jiangnan.edu.cn (Q.C.); qfflast@sina.com (F.Q.); 2 School of Food Science and Technology, Jiangnan University, Wuxi 214122, China

**Keywords:** soybean soluble polysaccharide, acidic milk drinks, ultrafiltration, molecular weight, protein, stabilization

## Abstract

Soybean Soluble Polysaccharide (SSPS) is a natural anionic polysaccharide with protein content extracted from soybean residue. However, the impact of molecular weight and degree of esterification (DE) of soybean polysaccharides on protein stabilization remains a topic of debate. This study aimed to clarify the composition, macromolecular structure, and protein stabilization mechanism of SSPS and its various fractions with differing DEs and molecular weights (MWs). Nine polysaccharide fractions were isolated from three types of SSPSs with varying DEs and MWs using membrane ultrafiltration treatment. The analysis of monosaccharide composition and protein content reveals that the first component of soybean polysaccharides with high (847 kDa) molecular weight and low DE(SSPS20I) possesses the highest (7.25%) concentration of galacturonic acid (GalA) and a lower (0.83%) protein content compared to high-esterification SSPS. Meanwhile, the analysis of amino acids revealed that glutamic acid and aspartic acid were the primary amino acids across all protein components. It was also evident that alkaline treatment influenced the amino acid composition of SSPS. Atomic Force Microscopy (AFM) further substantiated that the components of SSPS exhibit distinct morphological and structural characteristics. The effects of SSPS fractions on the stability of Acidic Milk Drinks (AMDs) were investigated and evaluated using LUMi-Sizer. The results suggest that SSPS20I provided better stabilization in AMDs. This work establishes critical structure–property correlations, revealing that both DE and MW govern SSPS stabilization efficacy through synergistic effects of electrostatic repulsion, steric hindrance, and interfacial adsorption capacity.

## 1. Introduction

Soluble soybean polysaccharide (SSPS) is an acidic, water-soluble polysaccharide with a pectin-like structure, typically extracted and refined from the byproducts of soybean protein isolation processes, such as soymilk and tofu production [1]. SSPS is thought to contain polysaccharide–protein conjugates and exhibits a comb-like architecture, which consists of a rhamnogalacturonan and homogalacturonan backbone with side chains of β-1,4-galactan and α-1,3- or α-1,5-arabinan [2,3]. Owing to its unique molecular structure and pectin-like functional characteristics, SSPS has attracted considerable interest from both academia and the food industry, particularly for its ability to stabilize proteins in acidic environments such as those found in acidified milk drinks (AMDs). As a dietary fiber derived from plant-based materials, SSPS holds promise not only for its emulsifying and stabilizing roles but also for its biocompatibility, low allergenicity, and consumer acceptance in clean-label formulations. These additional attributes further enhance its potential as a sustainable and multifunctional food ingredient in modern food systems. Given its amphiphilic nature and branched configuration, SSPS can adsorb at protein–droplet interfaces and provide steric stabilization, effectively preventing protein aggregation and precipitation at low pH. This makes SSPS particularly valuable in applications such as yogurt drinks, fermented soy beverages [4], and fruit-based dairy emulsions, where phase separation under acidic conditions can compromise product quality and shelf stability. Moreover, SSPS does not significantly alter flavor profiles or mouthfeel, making it compatible with a wide range of beverage matrices.

Building upon this functional foundation, various studies have investigated how the structural properties of SSPS influence its stabilization capacity. For example, Nakamura developed a high-molecular-weight (MW) SSPS complex crosslinked by phosphoric acid and observed that this modified SSPS exhibited significantly different protein-stabilizing behavior compared to the native form [5]. The variation in functional properties was hypothesized to arise from differences in molecular architecture introduced by crosslinking. In another study, a series of SSPS samples with similar MW (396–489 kDa) but varying degrees of esterification (DE) was prepared by sequential acid and alkali treatments [6]. These samples were used to stabilize AMDs within a pH range of 3.7 to 4.9. Their results indicated that protein stabilization efficacy correlated with DE, suggesting a strong influence of methylation on the behavior of SSPS in acidic systems. However, contrary findings were reported by Cai et al. [7], where SSPS samples with lower DE exhibited superior protein stabilization at pH 3.0–3.5, irrespective of molecular weight. These discrepancies emphasize the complexity of SSPS functionality, likely arising from variations in composition and structure—such as molecular weight, protein content, DE, and branching—introduced by different extraction and modification methods, including enzymatic, thermal, and acid/alkaline treatments [6,8]. Thus, the preparation process critically influences SSPS properties and functional behavior. As such, even SSPS products with similar average MW or DE values may demonstrate divergent functionalities depending on these subtle compositional variations. Moreover, protein residues covalently or non-covalently linked to SSPS may influence its interfacial adsorption behavior and electrostatic interactions with casein micelles under low pH, which is currently underexplored in comparative studies. Prior studies [9,10,11] have reported that molecular weight, protein content, and DE are key factors affecting the ability of SSPS to stabilize proteins in acidic media. However, the interdependence of these variables makes it challenging to isolate the effect of any single factor. For instance, the influence of molecular weight on stabilization performance under controlled DE and structural composition conditions remains poorly understood. Furthermore, the role of other structural parameters (such as the ratio of main chain to side chains, the level of protein conjugation, and side chain composition) has not been comprehensively elucidated. Recent research also suggests that solution properties such as ζ-potential, particle size distribution, and SSPS–protein interaction forces (e.g., covalent bond, hydrogen bonding, hydrophobic interaction) may offer further insight into the stabilization mechanism [12,13]. However, integrated studies combining structural characterization and functional evaluation remain scarce, limiting our ability to define precise structure–function relationships for SSPS in AMD systems. Moreover, despite the commercial relevance of SSPS in stabilizing acidic dairy or plant-based beverages, there is limited consensus on what constitutes an “optimal” structure for maximal performance. The presence of arabinan and galactan side chains may influence both solubility and interfacial properties, while the esterification pattern could affect the binding affinity to proteins or fat globules. Clarifying these aspects will support both ingredient design and targeted application development.

Therefore, this study aimed to clarify the composition, macromolecular structure, and protein stabilization mechanism of SSPS and its various fractions with differing DEs and MWs. Three commercial SSPS products were fractionated by ultrafiltration into distinct molecular weight distributions. Each SSPS and its fractions were evaluated for their capacity to stabilize milk proteins in acidic environments. Comprehensive analyses were conducted to determine their composition (including molecular weight, monosaccharide composition, DE, protein content, and amino acid composition), structural characteristics (via atomic force microscopy), and solution properties (ζ-potential, particle size distribution, and dispersion stability assessed by LUMi-Sizer). By correlating these structural and compositional parameters with protein stabilization performance, this study aims to provide a mechanistic understanding of how SSPS functions in acidic dairy systems. The findings are expected to support the rational design of next-generation SSPS-based stabilizers for use in functional beverages, particularly those formulated for health-conscious or plant-based markets, and also aid in setting specification criteria for SSPS used in the food industry.

## 2. Materials and Methods

### 2.1. Materials

Three commercial SSPS products, including SSPS20 (MW 475 kDa, DE 20%), SSPS60 (MW 483 kDa, DE 60%), and SSPSL20 (MW 395 kDa, DE 20%), were supplied by Jinjing Co., Ltd. (Pingdingshan, China). Ultrapure water was used in the preparation of all solutions. All chemicals used were of analytical grade.

### 2.2. Fractionation of SSPS

**Figure A1:**
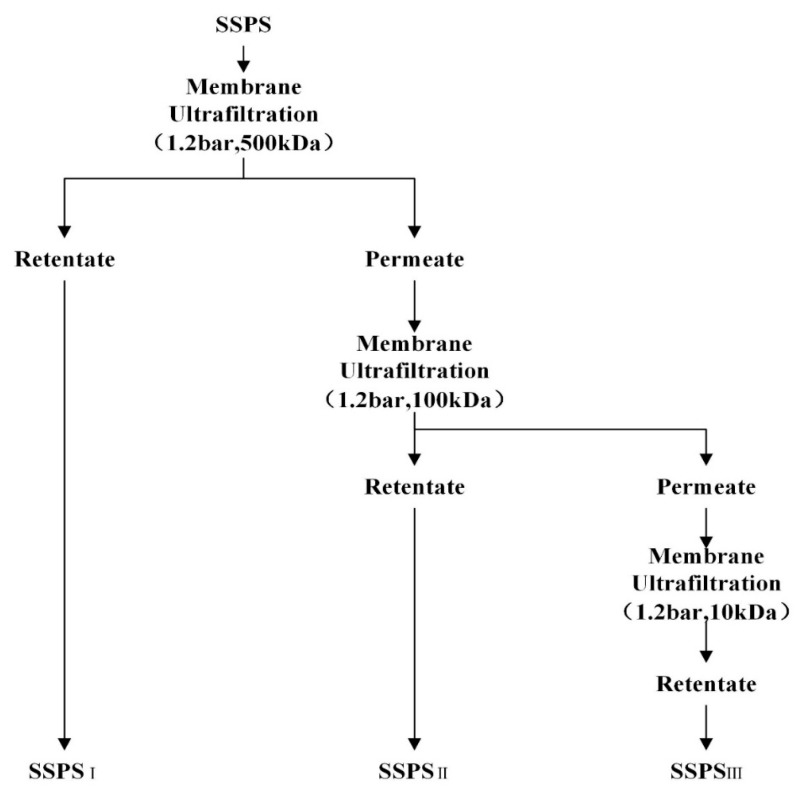
The procedure of SSPS fractionation by membrane ultrafiltration.

Different fractions of SSPS were prepared according to a previous work with some reasonable modifications [14]. As shown in Figure A1, the SSPS solution was ultra-filtrated successively by tangential flow filtration membranes with molecular weight cut-off of 500 kDa, 100 kDa, and 10 kDa (Pellicon^®^2, Merck Millipore Co., Ltd, Darmstadt, Germany). Three fractions (SSPSI, SSPSII, SSPSIII) obtained from the SSPS solution were concentrated to small volumes and also precipitated with four volumes of absolute ethanol for 24 h at 4 °C. The precipitates were collected by centrifugation at 5000× *g* for 20 min, and dried at 60 °C to obtain different fractions of SSPS.

### 2.3. Monosaccharide Composition

Monosaccharide composition analysis of SSPS fractions was carried out according to the method described by Tang [14]. In brief, 15 mg of the SSPS fractions were hydrolyzed by 0.5 mL of 12 M H_2_SO_4_ at 120 °C for 6 h in the thick-walled pressure bottle. The solutions were centrifuged at 6000 rpm for 10 min. The supernatant liquid was diluted 200 times. The sample solution was filtered through a 0.45 μm filter. The injection volume was 20 μL. The analysis was carried out on ion chromatography (Thermo, Dionex™ ICS-5000+, Santa Clara, CA, USA) equipped with a PA20 column (3 mm × 150 mm) (Thermo Scientific™ Dionex™ CarboPac™, Santa Clara, CA, USA). The mobile phase was 250 mM NaOH and 1 M CH_3_COONa. The flow rate was 0.5 mL/min.

### 2.4. Measurement of Molecular Weight and Distribution

The method was performed with reference to the previous work [15]. The molecular weight and distribution of SSPS were determined by high-performance gel filtration chromatography (HPGFC) using a Waters 2695 instrument equipped with a 2410 refractive index detector and a 2998 PDA detector. A TSK-gel-5000PWXL column (7.8 mm × 300 mm; Tosoh Co., Ltd., Tokyo, Japan) was calibrated with dextran standards (MW 5–2000 kDa) and eluted with 0.1 M phosphate buffer (pH 6.8) at a flow rate of 0.6 mL/min. SSPS samples (10 mg/mL) were dissolved in 0.1 M phosphate buffer (pH 6.8), filtered through a 0.45 μm filter, and injected into the HPGFC system (30 μL). The weight average molecular weight (MW) of SSPS was calculated and analyzed by Empower software (Version 2.0, Waters).

### 2.5. Zeta Potential and Particle Size Measurement

The zeta potential and particle size of SSPS solutions (1 mg/mL) and casein particles coated with SSPS in AMDs were determined at 4 pH Nano-Zetasizer (Malvern Instrument, Malvern, UK) at 25 °C, which was analyzed using Nano-Zetasizer software (version 7.13). AMDS solutions were diluted 100 times with citrate phosphate buffer (pH 4). All measurements were performed in at least triplicate, which were analyzed using Zetasizer software.

### 2.6. Rheological Analysis

The viscosity and rheological behavior analysis of the SSPS fractions were performed at shear rates from 0.1 to 100 s^−1^ with a rheology meter (HAKKE) equipped with plate geometry (P-60). The gap was 0.1 mm, and the temperature was controlled at 25 °C. To prevent moisture evaporation, a layer of low-viscosity silicone oil is applied to the surface of the exposed solution.

The rheological data for SSPS and SSPS fraction solutions were fitted to the power law model as follows:

τ = Kγ*^n^* (1)

τ: the shear stress; K: the consistency index (Pa·S*^n^*); γ: the shear rate; *n*: the flow behavior index. The values of n and K were obtained by regression of the double logarithmic plot [16].

### 2.7. Amino Acid Analysis

Amino acid analysis was performed after acid hydrolysis (6 M HCl, 0.1% phenol, 110 °C, 24 h) under an inert atmosphere [17]. The hydrolysates were filtered and analyzed by HPLC with pre-column derivatization. An Agilent 1100 HPLC system equipped with a Hypersil ODS column (4 mm × 250 mm, 5 μm) and a variable wavelength detector was used. Calibration was performed using amino acid standards, and an internal standard was added to each sample to ensure recovery and quantification accuracy.

### 2.8. Atomic Force Microscopy

The 1 mg/mL SSPS fraction water solution was prepared and diluted to 1–5 μg/mL using deionized water and 2 mM Tween-20 aqueous solution. The 2 μL of the diluted samples were immediately dropped onto freshly cleaved mica surfaces and dried in an enclosed Petri dish for 5 h [18]. The images of the Atomic force microscope (AFM) were obtained in the air at 20 °C and scanned in tapping mode using a silicon nitride cantilever (Bruker, Billerica, MA, USA) with a nominal radius of the pyramidal tip of 2 nm. Images of SSPS were obtained by the Nanoscope Analysis software (version 1.70, Bruker, Billerica, MA, USA).

### 2.9. Preparation of AMDs

Following a previously reported approach with modifications, the preparation method for AMDs was developed to address the requirements of the present investigation [7]. The reconstituted milk (8% *w*/*w*) was prepared by dispersing skim milk powder in the ultrapure water at 40 °C. Meanwhile, the SSPS samples (0.8% *w*/*w*) were obtained by dissolving SSPS in ultrapure water at 50 °C under stirring. Then skim milk dispersion and SSPS solution were mixed in a 1:1 ratio and stirred for 20 min to obtain the 100 mL mixed sample with a final protein concentration of 2 wt% containing 0.4 wt% SSPS. The pH of the samples was adjusted to 4.0 by 10% (*w*/*w*) citric buffer solution. The samples were homogenized with a PandaPLUS 2000 homogenizer (GEA, Milanese, Italy) at 40 bar for 5 min. The final samples were stored at 4 °C.

### 2.10. Measurement of AMDs Stability by LUMi-Sizer

The stabilities of AMDs stabilized by SSPS were measured with the stability analyzer LUMi-Sizer (L.U.M. GmbH, Berlin, Germany), which is a multi-sample analytical instrument employing centrifugal sedimentation to accelerate the occurrence of instability phenomena such as sedimentation, flocculation, or creaming [8]. The instrument can measure the interface between particle-free liquid and suspension/sediment of 12 samples simultaneously during centrifugation using a rectangular polycarbonate cuvette [19]. The cuvettes are fixed in the instrument, while parallel NIR-light is passed through the cuvettes, can simultaneously measure the intensity of transmitted light as a function of time and position over the entire sample length during the centrifugation [8]. AMDs (0.4 mL) stabilized by SSPS and SSPS fractions were filled into cuvettes. The instrument parameters used for measurement were set as follows: temperature, 25 °C; centrifugation speed, 4000 rpm; time interval, 10 s; test time, 3600 s. Each analysis was obtained in triplicate, and each result was shown as a mean ± standard deviation.

### 2.11. Statistical Analysis

Analytical data were obtained from three replicates and reported as mean ± SD. Tukey’s test (SPSS Inc., Tokyo, Japan) was used for statistical analysis. A *p*-value of less than 0.05 indicated a significant difference.

## 3. Results and Discussion

### 3.1. Molecular Weight and Particle Size of SSPS

**Figure 1:**
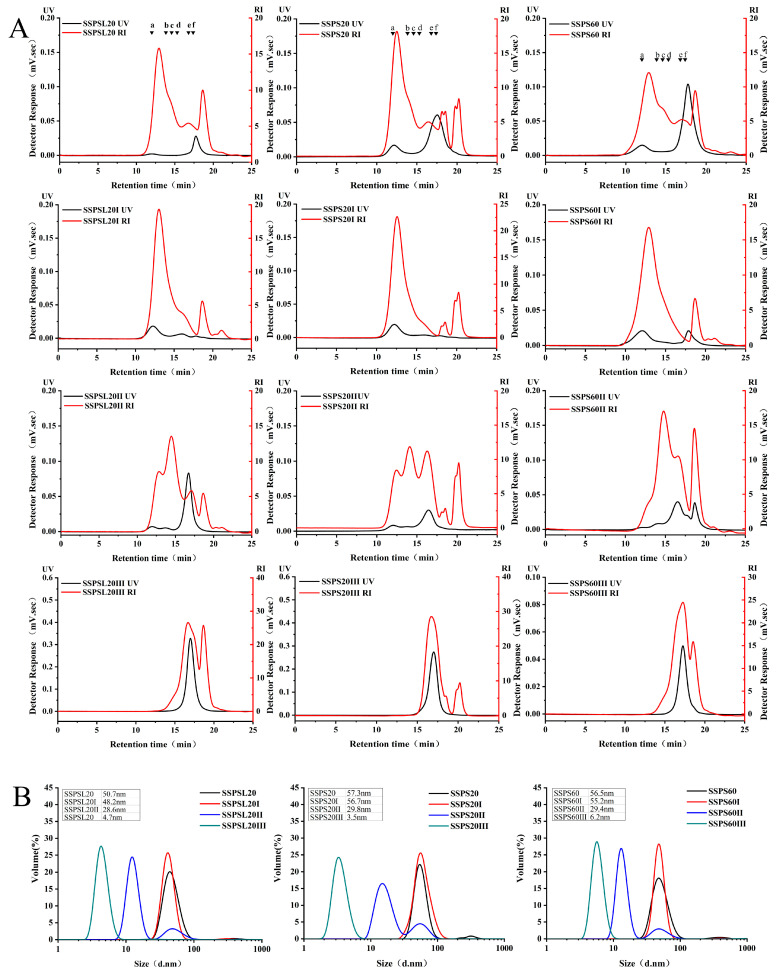
(**A**): Molecular weight distribution of original SSPS and different fractions. Red line, refractive index (RI) detection; black line, UV detector at 280 nm. Molecular masses (Da) were calculated using standard pullulans (a, 2,000,000 Da; b, 300,600 Da; c, 135,350 Da; d, 64,650 Da; e, 13,050 Da; f, 5250 Da); (**B**): Particle size distribution of original SSPS and different fractions.

The MW and particle size results of SSPS and their fractions are presented in Table 1 and Figure 1. As shown in Table 1 and Figure 1, the MW of SSPS20 and SSPS60 are similar, as are those of their fractional components. The molecular weight of SSPSL20 and its fractions is relatively low. Moreover, the content of the SSPS20I, SSPS60I, and SSPSL20I fractions, with a molecular weight above 500 kDa, is significantly higher than that of SSPS20, SSPS60, and SSPSL20. In contrast, the content of the SSPS20III and SSPS60III fractions with a molecular weight above 500 kDa is 0%. These findings indicate that the purity of the components across the three different molecular weight ranges, obtained by ultrafiltration treatment, is markedly improved compared to their state before ultrafiltration. This implies that the SSPS was successfully fractionated into three distinct components. Table 1 also displays the average particle size results of three types of SSPS with varying DE and MW at pH 4. All three types of SSPS were successfully separated into three fractions. The average particle size of SSPS20 and SSPS20I is similar, approximately 57 nm. The results show that the dominant fraction of SSPS is SSPSI, and the results for SSPS60 are similar to those for SSPS20. The smallest average particle size was observed for SSPSL20 and its fractions. Similar results were reported in a previous paper [17].

### 3.2. Monosaccharide Compositions and Protein Content

The intricate structure of SSPS has been elucidated through a methodical process of enzymatic degradation [20]. The primary backbone of SSPS consists of Galacturonan (GN) and Rhamnogalacturonan (RG), while the highly branched neutral sugar chains are constituted by Galactan and Arabinan [5]. Consequently, the monosaccharide composition and the Ara/Rha ratio can serve as indicators of the ratio between the main chain and the branched chain in SSPS. When considered in conjunction with protein content and DE, these factors can provide insights into the structure of SSPS. Table 2 presents the monosaccharide compositions and protein contents of various SSPS fractions. Nine monosaccharides were identified in the three types of SSPS and their respective fractions. A comparative analysis of the monosaccharide composition, Ara/Rha ratio, protein content, and DE of SSPS20 and its three ultrafiltration fractions revealed significant differences among the three fractions of SSPS with low DE. As the molecular weight decreased, the content of Rhamnose and galacturonic acid (GalA) decreased sharply, while the Ara/Rha ratio consistently increased, and the protein content rose significantly. This suggests that the three components within SSPS exhibit considerable structural differences. In comparison to components SSPS20II and SSPS20III, component SSPS20I possesses a longer main chain structure and relatively lower protein content. A comparison of the monosaccharide composition, protein composition, molecular weight, and DE of SSPS20, SSPS60, SSPSL20, and their fractions indicates that alkaline treatment and acid extraction of SSPS could lead to a loss of covalently attached protein and molecular weight. This may be because SSPSI possesses a longer main backbone, whereas SSPSIII contains some free protein. The protein in SSPSI is a covalently attached, formed by combining a protein with carbohydrate side chains [10]. The protein content of SSPS20II was found to be higher than that of SSPS60II, likely because the content of fractions greater than 500 kDa in SSPS20II exceeded that in SSPS60II. Liang has revealed that alkali treatment could affect the monosaccharide composition of pectin, with breakage of the pectin main chain by alkali treatment potentially leading to an increased ratio of (Gal + Ara)/Rha [21]. These findings further underscore that different fractions of SSPS exhibit considerable structural differences, not only in molecular weight but also in the ratio of the main chain to neutral sugar side chain and in protein content. Therefore, it can be inferred that the composition and content of these three fractions may influence the overall properties of SSPS, particularly its ability to stabilize proteins under acidic conditions.

### 3.3. Zeta Potential

**Figure 2:**
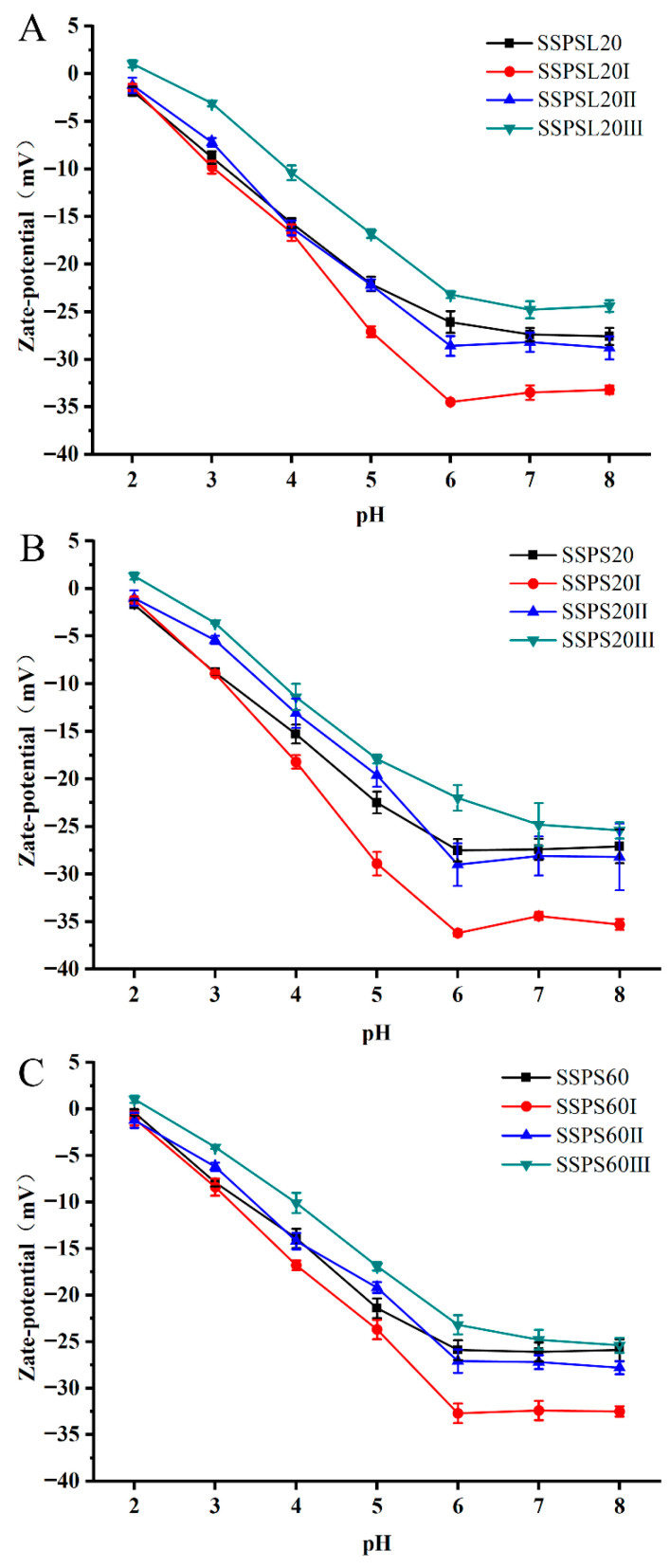
Zeta potentials of SSPS (**A**): SSPSL20; (**B**): SSPS20; (**C**): SSPS60 and its fractions in aqueous solutions at pH 2–8 (*p* < 0.05).

The interplay between attractive and repulsive forces among colloidal particles plays a pivotal role in determining the stability and physicochemical properties of dispersions [22]. The zeta potentials of SSPS20, SSPS60, SSPSL20, and fractions with varying MW across a pH range of 2 to 8 are depicted in Figure 2. Given the variability in the GalA content across different fractions, zeta potential measurements were used to assess the surface charge of SSPS solutions. Within the tested pH ranges, the absolute zeta potential values of SSPS20 and its fractions were higher than those of SSPS60, while being similar to those of SSPSL20. As pH increased from 2 to 8, the zeta potential of SSPS20I decreased significantly from −1.1 mV to −35.3 mV, which can be attributable to the ionization of carboxylic groups. SSPS20I solutions exhibited larger zeta potentials, likely due to the higher GalA content in SSPS20I compared to other fractions and SSPS60I (refer to Table 2). These results indicate that the number of negative charges on SSPS significantly affects its dispersion and stabilization properties.

### 3.4. Amino Acid Composition Analysis

The protein content of SSPS affects not only its surface activity (including emulsification and emulsion stability, foaming and foam stability) but also its ability to stabilize proteins under acidic conditions. Therefore, we analyzed SSPS20, SSPS60, SSPSL20, and their fractions with different MW. As shown in Table 3, SSPS60 exhibited higher amino acid contents than SSPS20 and SSPSL20, with the SSPSIII fractions showing the highest levels across all three samples. This result was consistent with the protein content data in Table 2. SSPS60I had a significantly higher protein content than both SSPS20I and SSPSL20I. It was reported that the alkaline treatment of SSPS leads to the loss of covalently bound protein [6]. The three fractions and the raw SSPS material exhibited marked differences in their specific composition. In all fractions, glutamic acid and aspartic acid were the dominant amino acids. The glutamic acid content of SSPSIII was much higher than that of SSPSI and SSPSII, and also higher than that of the raw material SSPS, regardless of whether it was SSPS20, SSPS60, or SSPSL20. This result is consistent with the findings of Nakamura [17]. In SSPS20III, the levels of Ser, His, Gly, Thr, Arg, Met, and Lys were substantially higher than those in the other two fractions and were similar to the levels in the raw SSPS material. The levels of other amino acids were comparable. In the fractions of SSPS60, SSPS60III exhibited much higher levels of Ser, His, Gly, Thr, Arg, Met, and Lys than the other two fractions, even though SSPS60II had a lower overall protein content. This was likely because free protein in SSPS was retained in the SSPSIII fraction during membrane ultrafiltration [23]. These results confirm the previous conclusion that the three fractions have distinct structures. Moreover, the binding modes between protein and polysaccharide may also differ. The structures of these fractions differed significantly, consequently leading to different properties. This finding indicated that the protein component in SSPS may adversely affect the protein-stabilizing capability of SSPS. Alkaline treatment-induced de-esterification not only reduces the DE—thereby increasing the number of negative charges density of SSPS—but also diminishes its protein content. More definitive conclusions necessitate further in-depth investigation.

### 3.5. Apparent Viscosity Analysis

**Figure 3:**
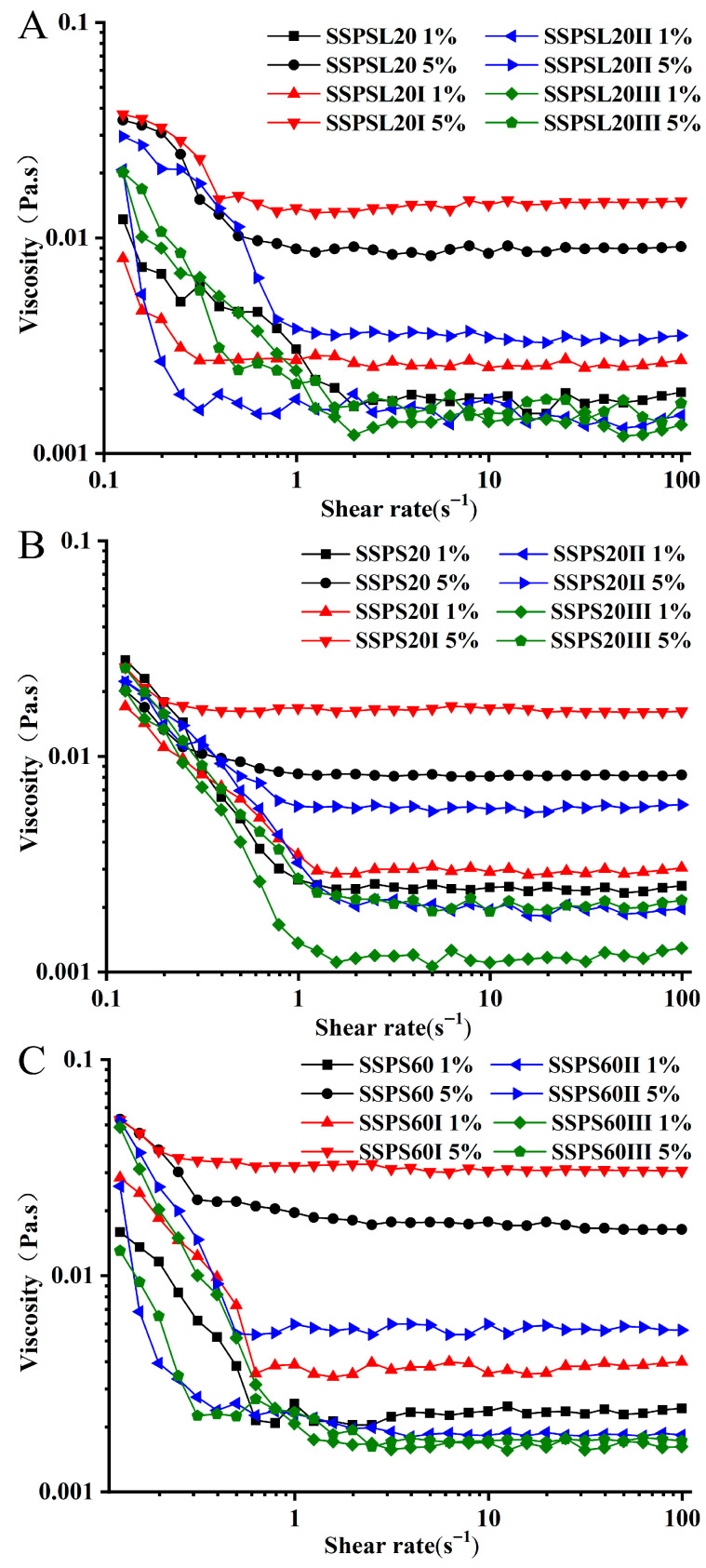
The steady shear viscosity of SSPS (**A**): SSPSL20; (**B**): SSPS20; (**C**): SSPS60 and SSPS fractions at different concentrations on the shear rate.

Figure 3 shows the apparent viscosity of three different types of SSPS and their fractions, at various concentrations in water. The results indicate that the apparent viscosity of all SSPS fractions increases with concentration; however, the values remain low even at higher concentrations. SSPS60 exhibited a higher apparent viscosity than SSPS20 and SSPSL20. The highest apparent viscosity among the samples was 0.0314 Pa s, observed for 5 wt% SSPS60I solution. The rheological property of viscosity in polymers is predominantly influenced by factors such as the polymer’s molecular mass and the rigidity of its molecular chains [16]. At a low shear rate (0.1–1 s^−1^), all SSPS fractions and solutions at different concentrations exhibited shear-thinning behaviors. A Newtonian plateau appeared at shear rates above 1 s^−1^, and this plateau broadened at higher concentrations of SSPS and SSPSI solutions. It is important to note that the shear-thinning behavior of SSPS in water differs from that of typical linear polymers. The apparent viscosity curves confirmed the shear-thinning behavior of SSPS and their fraction solutions, which followed the power-law model. As shown in Table 4, the flow behavior index (*n*) for 1% and 5% SSPS solutions and all fraction solutions was close to 1.0, indicating a near-Newtonian behavior. Consequently, acidic milk drinks stabilized with SSPS exhibit lower viscosity and a light taste without stickiness.

### 3.6. AFM Analysis of SSPS

**Figure 4:**
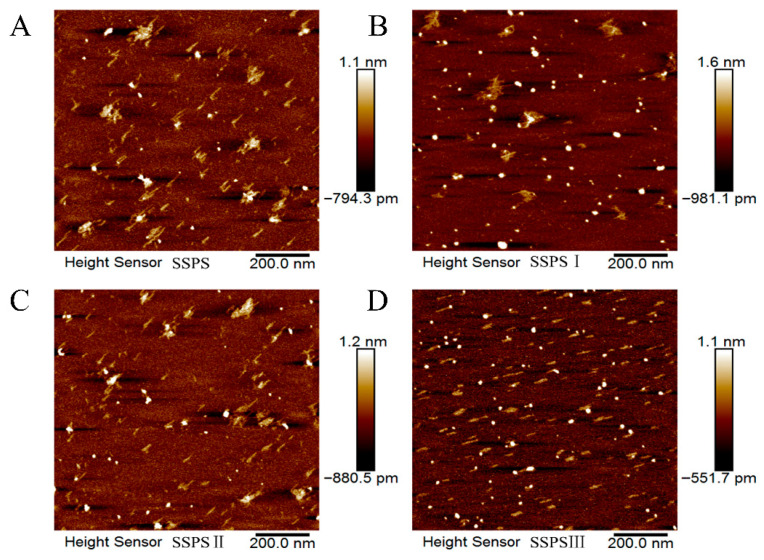
Image of SSPS (**A**): SSPS; (**B**): SSPSI; (**C**): SSPSII; (**D**): SSPSIII deposited from a 2 μg/mL solution with 2 mM Tween 20 (image size 1 μm × 1 μm).

To directly visualize the structural heterogeneity inherent in SSPS, we employed AFM to characterize its morphological and structural features. Figure 4 presents AFM images of SSPS and its fractions obtained by ultrafiltration. To prevent aggregation of SSPS chains during drying, the surfactant Tween 20 was added to the sample solutions to facilitate structural analysis. As shown in Figure 4, SSPS molecules exhibit a star-like polymer morphology, with a height of 0.5–1.0 nm and a maximum dimension of less than 100 nm. This observation is consistent with the findings reported by Nakamura et al. (2012) [5]. Comparative analysis of the AFM images revealed that the proportion of low- and medium-molecular-weight components was significantly lower in the SSPSI fraction than in the SSPSII fraction. The SSPSIII fraction exhibited a distinct morphology, with low-molecular-weight components exhibiting a chain-like structure, which differed markedly from the high-molecular-weight SSPS. Combined with the monosaccharide composition results, these chain-like structures likely represent fragmented main chains and side chains of SSPS. The SSPSIII fraction, which lacks the intact SSPS structure, demonstrates ineffective adsorption onto the positively charged surface of casein particles during the stabilization of Acidified Dairy Mixtures (ADMs). Furthermore, owing to the absence of complete side chains architectures, it fails to deliver adequate steric hindrance, a finding consistent with subsequent experimental results. The highlighted regions in Figure 4 represent aggregated SSPS structures that were not fully dispersed by the surfactant. The height of these spherical aggregates was greater than that of the star-like polymers due to intramolecular aggregation. Additionally, spherical particles with diameters ranging from approximately 20 to 40 nm were predominantly observed. These results indicate significant differences in particle diameter among the various fractions. These AFM observations are consistent with the particle size measurements.

### 3.7. Physical Stability Analysis of AMDs by LUMi-Sizer

**Figure 5:**
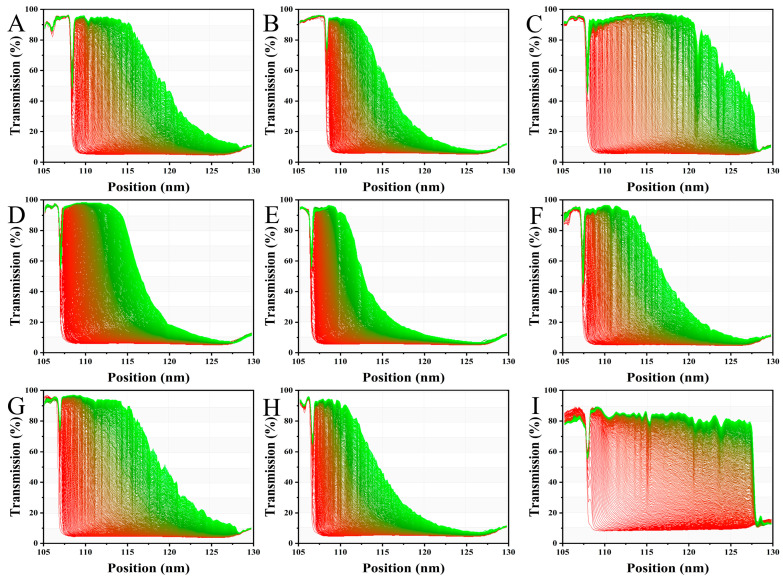
Transmission profiles of SSPS-stabilized AMDs measured by a LUMi-Sizer. (**A**): SSPSL20; (**B**): SSPSL20I; (**C**): SSPSL20II; (**D**): SSPS20; (**E**): SSPS20I; (**F**): SSPS20II; (**G**): SSPS60; (**H**): SSPS60I; (**I**): SSPS60II.

**Figure 6:**
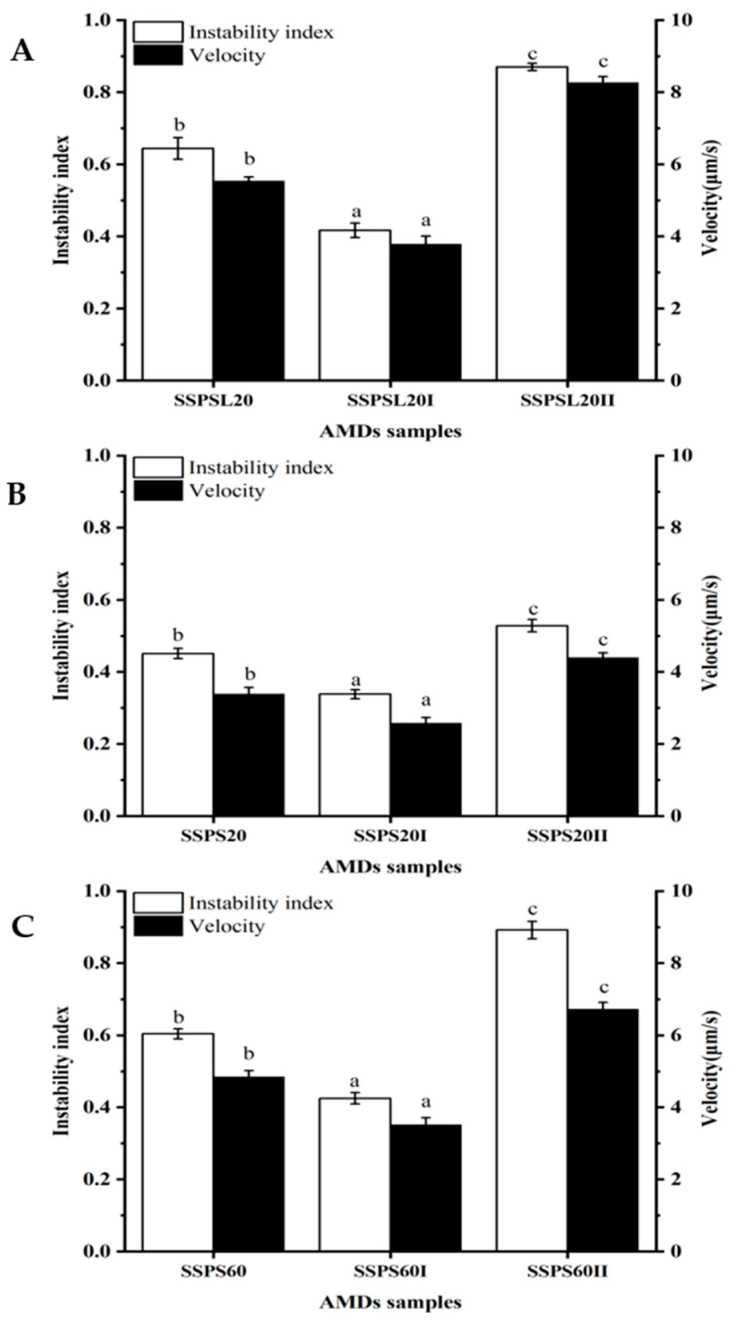
Instability indices and sedimentation velocities of AMDs stabilized by SSPS (**A**): SSPSL20; (**B**): SSPS20; (**C**): SSPS60 and SSPS fractions at pH 4 measured by LUMiSizer. Different letters indicate a statistically significant difference (*p* < 0.05).

The stability of AMDs stabilized by SSPS20, SSPS60, SSPSL20, and their fractions was assessed using the LUMi-Sizer analyzer via an accelerated destabilization test. This method, which utilizes centrifugal force under accelerated conditions, is highly effective for predicting long-term dispersion stability and enabling the early detection of precipitation. Figure 5 shows the evolution of transmission profiles for AMDs stabilized by SSPS and its fractions. The high light transmission at approximately 106 mm signifies the position of the meniscus (liquid level), whereas the region of low light transmission near 129 mm indicates the bottom of the sample cell. The temporal evolution of the transmission profiles reflects the sedimentation kinetics of the casein particles during centrifugation. The initial profile (T = 0) represents the start of centrifugation, indicating low light transmission and a stable, homogeneous dispersion of casein particles. The progressive increase in light transmission over time is characteristic of the sedimentation process. A more rapid increase in the overall transmission signal over time during centrifugation suggests greater instability in the dispersions. AMDs stabilized by SSPS20, SSPS60, SSPSL20, and their respective fractions exhibited significant differences in stability (Figure 5). SSPS20 exhibited higher stability than SSPS60 and SSPSL20, which can be attributed to its higher MW and lower degree of DE. AMDs stabilized by SSPS20I exhibited high stability, attributable to their higher GalA content and greater absolute zeta potential value compared to other SSPS types and fractions. In contrast, SSPSIII did not stabilize the ADMs, indicating that SSPSI is the predominant fraction responsible for stabilizing casein particles in AMDs. This stabilization is primarily due to a combination of strong electrostatic repulsion and steric hindrance. The instability index and sedimentation velocity were calculated using SEPView (version 6.0) software, which is designed for analyzing the sedimentation behaviors of colloidal dispersions such as AMDS. Higher values of both the instability index and sedimentation velocity indicate lower stability. As shown in Figure 6, SSPS20I-stabilized AMDs exhibited the lowest instability index (0.33), indicating the highest stability among the samples. The instability indices for SSPS20 and SSPS20II were 0.44 and 0.52, respectively. Figure 6 also compares the sedimentation velocity of AMDs stabilized by SSPS20, SSPS20I, and SSPS20II, which were measured as 3.39 μm/s, 2.58 μm/s, and 4.40 μm/s, respectively. These results demonstrate that SSPS20I possesses a strong capability to prevent casein particle aggregation, primarily owing to its high molecular weight and large absolute zeta potential. This finding differs from those reported in previous studies [7], highlighting the distinctive structural and functional properties of SSPS20I observed in this work. Furthermore, the high-molecular-weight fraction SSPSI plays a predominant role in stabilizing casein particles in AMDs.

### 3.8. Particle Size and Zeta Potential of Casein Particles Coated by Different Fractions of SSPS

**Figure 7:**
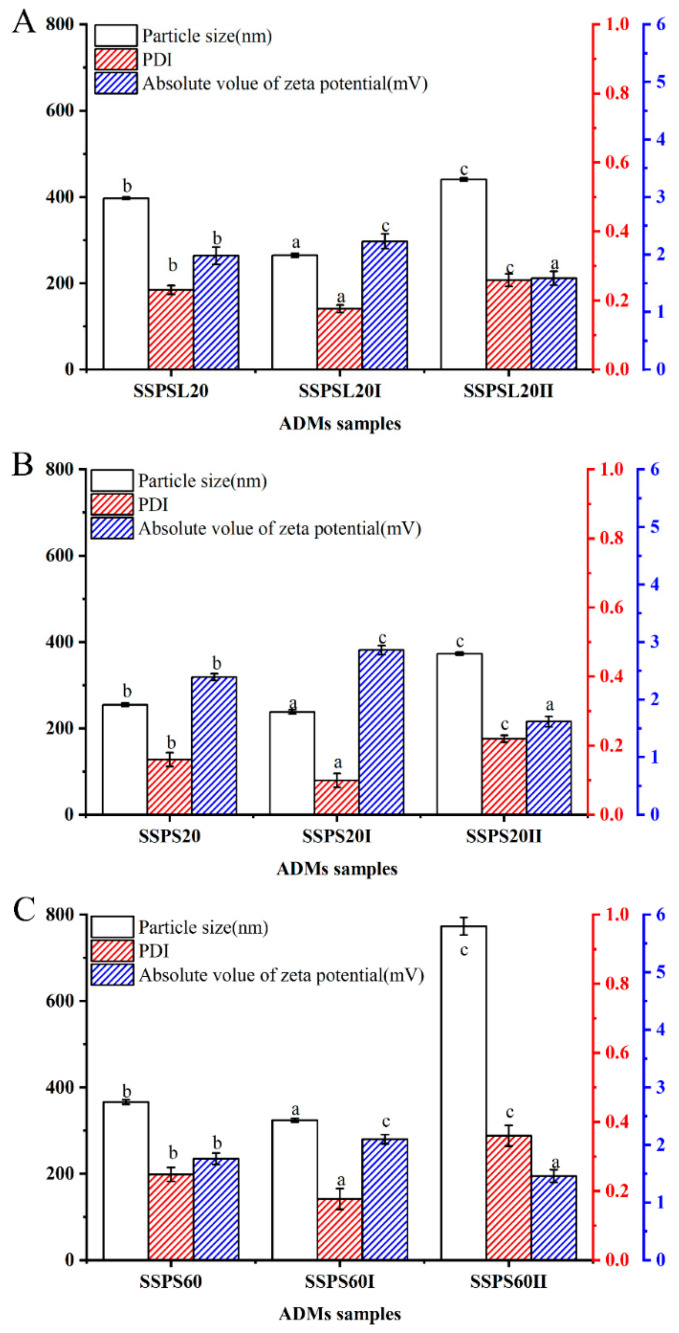
The average particle sizes, absolute value of zeta potentials, and value of PDI of AMDs (**A**): SSPSL20; (**B**): SSPS20; (**C**): SSPS60. Data not sharing a common letter are significantly different (*p* < 0.05).

This study found that different fractions of SSPS20, SSPS60, and SSPSL20 exhibited distinct particle sizes and zeta potentials, among which SSPS20I demonstrated the highest capability to stabilize AMDs. As described by Stokes’ law, a fundamental model for sedimentation stability, the stability of colloidal dispersions is governed by the particle size and the viscosity of the continuous phase [24]. To investigate the stabilization mechanism of AMDs by various SSPS fractions, the average particle size and zeta potential of the casein particles in the AMDs were measured. As shown in Figure 7, the average particle size of AMDs stabilized by SSPS20 was smaller than that of those stabilized by SSPS60 and SSPSL20. Additionally, the absolute value of the zeta potential of AMDs stabilized by SSPS20 was higher than that of those stabilized by SSPS60. The average particle size of AMDs stabilized by SSPS20I was 238 nm, with a Polydispersity Index (PDI) value of 0.1 and a zeta potential absolute value of 2.86 mV. These results suggest that casein micelles were well dispersed without aggregation. The absolute zeta potential values of AMDs stabilized by SSPS60 and its high-DE-value fractions were lower than those of AMDs stabilized by SSPS20 and SSPSL20. Coating the surface of casein particles with low-DE SSPS resulted in casein-SSPS complex particles with a high absolute zeta potential. This enhanced electrostatic repulsion between the complex particles thereby inhibited the phase separation of casein particles in AMDs [25]. For SSPS60 and SSPSL20, only AMDs stabilized by SSPSL20I had an average particle size below 300 nm. This further confirms that SSPS20I possesses a strong ability to prevent casein particle aggregation, primarily owing to its high molecular weight and large absolute zeta potential. It should be noted that a decrease in particle size does not universally promote stability; numerous studies have reported that in AMDs, excessively small particles under certain conditions may exhibit increased sedimentation due to reduced electrostatic repulsion and other factors [20].

### 3.9. Protein Dispersing and Stabilizing Mechanism of AMDs by Using SSPS

**Figure 8:**
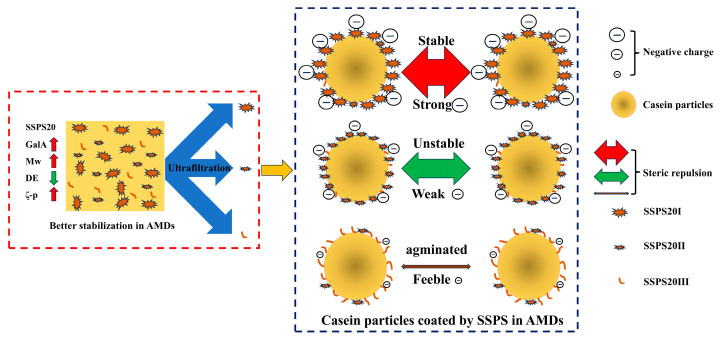
Schematic illustration of the casein particles stabilized by SSPS fractions in AMDs.

As illustrated in Figure 8, based on the compositional and structural differences among SSPS20I, SSPS20II, and SSPS20III, along with the results from physical stability analysis, it can be inferred that SSPS20I (characterized by a high MW and low DE) plays the most critical role in stabilizing AMDs. The stability of AMDs is highly dependent on both the DE and MW of the SSPS-based stabilizers. The negatively charged galacturonic acid backbone of SSPS adsorbs onto positively charged surface patches of casein particles, while neutral side chains composed of galactan and arabinan extend into the aqueous phase to form a thick hydrated layer. This configuration stabilizes the casein particles through combined electrostatic and steric repulsion forces [7]. Interestingly, the stabilization mechanism of SSPS shows remarkable similarity to that of pectin, a widely studied polysaccharide stabilizer. Investigations on pectin have revealed that below pH 5.0, electrostatically charged segments of the pectin chains adsorb onto positively charged surfaces of casein particles, while the neutral segments protrude into the aqueous phase to form extended loops. This configuration effectively impedes particle aggregation through steric hindrance, thereby enhancing colloidal stability of the system [26,27]. Similar results have also been reported for milk protein particles stabilized by water-soluble polysaccharides derived from peas [28].

## 4. Conclusions

In this study, nine polysaccharide fractions were sequentially isolated from SSPS20, SSPS60, and SSPSL20 using membrane ultrafiltration technology. The results demonstrated that these fractions, with distinct MWs and degrees of esterification, exhibited significant differences in chemical composition, physicochemical properties, structures, and conformations. Among all fractions, SSPS20I, characterized by a high molecular weight and a low degree of esterification, exhibited the highest efficacy in stabilizing acidified milk drinks (AMDs). This superior performance was attributed to its high GalA content, pronounced negative zeta potential, and larger particle size, which enhanced electrostatic repulsion and steric hindrance between SSPS-coated casein particles. Overall, these findings clarify the structural and functional differences among SSPS fractions induced by alkaline treatment and advance our understanding of the stabilization mechanisms in AMDs. Importantly, the results indicate that targeted selection or modification of SSPS fractions—based on molecular weight and chemical composition—represents an effective strategy for designing stabilizers with tailored performance for acidic dairy beverages. From an industrial perspective, such insights could guide the production of SSPS ingredients with optimized stability, texture, and sensory properties. Future studies should further evaluate the feasibility of scale-up, cost-effectiveness, and potential sensory impacts to facilitate the practical application of SSPS-based stabilizers in the dairy industry.

## Figures and Tables

**Table 1:** MW, MW distribution range and particle size of original SSPS and different fractions.

Sample	MW (kDa)	Molecular Weight Distribution Range (%)				Particle Size (nm)
		>500 kDa	500~100 kDa	100~10 kDa	<10 kDa	
SSPS20 *	475	54.12	15.67	14.39	15.82	57.3 ± 0.7
SSPS20I	847	72.19	15.04	1.98	10.79	56.7 ± 0.9
SSPS20II	380	18.65	31.01	30.47	19.87	29.8 ± 0.7
SSPS20III	44	0.00	2.15	81.88	15.97	3.5 ± 0.1
SSPS60 *	483	46.90	18.45	16.63	18.02	56.5 ± 0.6
SSPS60I	859	73.49	14.61	2.17	9.74	55.2 ± 0.6
SSPS60II	369	7.27	52.76	20.08	19.90	29.4 ± 0.5
SSPS60III	45	0.00	4.53	73.80	21.67	6.2 ± 0.1
SSPSL20 *	395	42.15	19.87	19.01	18.97	50.7 ± 0.5
SSPSL20I	716	67.92	17.42	2.36	14.20	48.2± 0.6
SSPSL20II	305	16.46	42.19	22.48	18.87	28.6 ± 0.3
SSPSL20III	41	0.00	3.58	69.26	27.16	4.7 ± 0.2

*: Original SSPS.

**Table 2:** Monosaccharide composition and protein content of different SSPS fractions.

Sample	Neutral Sugar (%)							GalA	GlcA	Ara/Rha	Protein (%)
	Fuc	Ara	Rha	Gal	Glc	Xyl	Man				
SSPS20	2.47	15.30	2.86	64.66	3.78	4.46	0.29	5.97	0.21	5.35	3.11 ± 0.30
SSPS20I	3.52	16.28	3.21	63.01	-	5.59	1.00	7.25	0.14	5.07	0.83 ± 0.11
SSPS20II	2.50	14.66	2.62	68.21	1.66	4.01	0.41	5.72	0.21	5.60	1.13 ± 0.08
SSPS20III	0.89	4.04	0.42	68.25	18.6	2.85	1.14	3.64	0.15	9.62	3.07 ± 0.12
SSPS60	3.90	17.13	4.28	58.84	1.06	1.72	7.65	5.04	0.73	4.00	3.80 ± 0.10
SSPS60I	5.67	18.85	4.06	56.61	-	-	8.1	6.08	0.63	4.64	2.15 ± 0.13
SSPS60II	2.58	19.17	4.12	64.85	0.49	0.50	4.33	3.50	0.45	4.65	0.69 ± 0.15
SSPS60III	2.25	9.93	1.78	77.63	1.02	3.64	0.39	2.81	0.54	5.58	4.23 ± 0.17
SSPSL20	3.29	16.37	4.15	63.79	2.07	4.51	0.20	5.33	0.29	3.94	2.70 ± 0.10
SSPSL20I	4.36	16.20	4.03	61.83	0.14	-	6.70	6.48	0.25	4.02	0.57 ± 0.10
SSPSL20II	2.14	15.96	3.34	70.67	0.18	3.47	0.25	3.83	0.16	4.78	0.80 ± 0.12
SSPSL20III	1.32	4.98	0.56	70.35	15.1	4.36	0.76	2.31	0.19	8.89	3.58 ± 0.15

Abbreviation: The symbol ‘-’ denotes a value that is negligible or very low. Fuc, Fucose; Ara, Arabinose; Rha, Rhamnose; Gal, Galactose; Glc, Glucose; Xyl, Xylose; Man, Mannose; GalA, galacturonic acid; GlcA, Glucuronic acid.

**Table 3:** Amino acid composition of original SSPS and different polysaccharide fractions.

Amino Acid (g/100 g)	SSPSL20	SSPSL20I	SSPSL20II	SSPSL20III	SSPS20	SSPS20I	SSPS20II	SSPS20III	SSPS60	SSPS60I	SSPS60II	SSPS60III
Asp	0.412	0.099	0.140	0.508	0.499	0.169	0.192	0.614	0.543	0.211	0.110	0.699
Glu	0.655	0.143	0.285	1.44	0.762	0.139	0.278	1.820	0.817	0.288	0.208	1.72
Ser	0.113	0.03	0.029	0.116	0.141	0.028	0.037	0.155	0.157	0.092	0.031	0.153
His	0.100	0.031	0.037	0.172	0.097	0.022	0.042	0.180	0.133	0.079	0.035	0.153
Gly	0.112	0.029	0.029	0.121	0.136	0.023	0.037	0.149	0.138	0.081	0.033	0.136
Thr	0.077	0.021	0.017	0.062	0.119	0.022	0.033	0.103	0.115	0.078	0.021	0.093
Arg	0.089	0.024	0.050	0.281	0.144	0.048	0.068	0.360	0.192	0.100	0.059	0.266
Ala	0.087	0.026	0.017	0.052	0.129	0.039	0.039	0.076	0.127	0.100	0.022	0.074
Tyr	0.048	0.012	0.011	0.04	0.068	0.008	0.009	0.042	0.041	0.028	0.011	0.055
Cys	0.002	0.000	0.003	0.003	0.001	0.000	0.001	0.001	0.005	0.002	0.002	0.002
Val	0.075	0.024	0.019	0.054	0.105	0.032	0.027	0.066	0.111	0.096	0.021	0.078
Met	0.013	0.003	0.002	0.009	0.018	0.003	0.001	0.022	0.007	0.004	0.004	0.014
Phe	0.057	0.018	0.016	0.058	0.090	0.024	0.023	0.077	0.083	0.067	0.017	0.065
Ile	0.057	0.018	0.015	0.056	0.080	0.021	0.019	0.069	0.086	0.077	0.016	0.077
Leu	0.086	0.029	0.016	0.053	0.130	0.004	0.028	0.060	0.136	0.129	0.021	0.078
Lys	0.131	0.039	0.046	0.220	0.184	0.005	0.057	0.280	0.221	0.134	0.044	0.226
Pro	0.107	0.032	0.072	0.333	0.281	0.089	0.106	0.509	0.275	0.082	0.037	0.334
Protein content	2.221	0.578	0.804	3.578	2.984	0.676	0.997	4.583	3.188	1.648	0.692	4.223

Abbreviation: Asp, Aspartic; Glu, Glutamic; Ser, Serine; His, Histidine; Gly, Glycine; Thr, Threonine; Arg, Arginine; Ala, Alanine; Tyr, Tyrosine; Cys, Cysteine; Val, Valine; Met, Methionine; Phe, Phenylalanine; Ile, Isoleucine; Leu, Leucine; Lys, Lysine; Pro, Proline.

**Table 4:** Rheological parameters of the power law equation for SSPS and SSPS fractions.

Sample	Concentration	K (Pa S*^n^*)	*n*	R^2^
SSPSL20	1%	0.0012 ± 0.0001	1.010 ± 0.0001	0.997
	5%	0.0082 ± 0.0001	1.022 ± 0.0001	0.999
SSPSL20I	1%	0.0020 ± 0.0001	1.062 ± 0.0001	0.999
	5%	0.0139 ± 0.0001	1.012 ± 0.0001	0.999
SSPSL20II	1%	0.0011 ± 0.0001	1.013 ± 0.0001	0.997
	5%	0.0054 ± 0.0001	1.012 ± 0.0001	0.999
SSPSL20III	1%	0.008 ± 0.0001	0.979 ± 0.0001	0.995
	5%	0.0018 ± 0.0001	0.987 ± 0.0001	0.996
SSPS20	1%	0.0020 ± 0.0001	0.996 ± 0.006	0.998
	5%	0.0083 ± 0.0002	0.985 ± 0.005	0.999
SSPS20I	1%	0.0022 ± 0.0001	0.993 ± 0.002	0.999
	5%	0.017 ± 0.0005	0.983 ± 0.007	0.999
SSPS20II	1%	0.0017 ± 0.0001	0.996 ± 0.008	0.997
	5%	0.0051 ± 0.0001	0.994 ± 0.006	0.999
SSPS20III	1%	0.0005 ± 0.0001	0.998 ± 0.003	0.997
	5%	0.0010 ± 0.0001	1.021 ± 0.0001	0.997
SSPS60	1%	0.0019 ± 0.0001	1.051 ±0.003	0.999
	5%	0.0196 ± 0.0002	0.959 ± 0.004	0.999
SSPS60I	1%	0.0019 ± 0.0001	1.061 ± 0.0001	0.999
	5%	0.0314 ± 0.0001	0.994 ± 0.0001	0.999
SSPS60II	1%	0.0025 ± 0.0001	1.010 ± 0.0002	0.999
	5%	0.0098 ± 0.0001	0.9912 ± 0.0004	0.999
SSPS60III	1%	0.0006 ± 0.0002	0.9944 ± 0.0001	0.999
	5%	0.0010 ± 0.0001	1.021 ± 0.0001	0.997

## Data Availability

The original contributions presented in the study are included in the article; further inquiries can be directed to the corresponding author.

## Abbreviations

The following abbreviations are used in this manuscript:

SSPS	Soybean Soluble Polysaccharide
AMDs	acidified milk beverages
DE	degree of esterification
MW	molecular weight
GalA	galacturonic acid

## References

[1] Maeda H., Nakamura A. (2009). Soluble Soybean Polysaccharide. Handbook of Hydrocolloids.

[2] Nakamura A., Furuta H., Maeda H., Takao T., Nagamatsu Y. (2002). Structural Studies by Stepwise Enzymatic Degradation of the Main Backbone of Soybean Soluble Polysaccharides Consisting of Galacturonan and Rhamnogalacturonan. Biosci. Biotechnol. Biochem..

[3] Nakamura A., Furuta H., Maeda H., Nagamatsu Y., Yoshimoto A. (2001). Analysis of Structural Components and Molecular Construction of Soybean Soluble Polysaccharides by Stepwise Enzymatic Degradation. Biosci. Biotechnol. Biochem..

[4] Niamah A.K., Sahi A.A., Al-Sharifi A.S.N. (2017). Effect of Feeding Soy Milk Fermented by Probiotic Bacteria on Some Blood Criteria and Weight of Experimental Animals. Probiotics Antimicrob. Proteins.

[5] Nakamura A., Fujii N., Tobe J., Adachi N., Hirotsuka M. (2012). Characterization and Functional Properties of Soybean High-Molecular-Mass Polysaccharide Complex. Food Hydrocoll..

[6] Xiong X., Zhao L., Chen Y., Ruan Q., Zhang C., Hua Y. (2015). Effects of Alkali Treatment and Subsequent Acidic Extraction on the Properties of Soybean Soluble Polysaccharides. Food Bioprod. Process..

[7] Cai Z., Wei Y., Guo Y., Ma A., Zhang H. (2020). Influence of the Degree of Esterification of Soluble Soybean Polysaccharide on the Stability of Acidified Milk Drinks. Food Hydrocoll..

[8] Cai Z., Wu J., Du B., Zhang H. (2018). Impact of Distribution of Carboxymethyl Substituents in the Stabilizer of Carboxymethyl Cellulose on the Stability of Acidified Milk Drinks. Food Hydrocoll..

[9] Cai Y., Cai B., Ikeda S. (2017). Stabilization of Milk Proteins in Acidic Conditions by Pectic Polysaccharides Extracted from Soy Flour. J. Dairy Sci..

[10] Liu J.R., Nakamura A., Corredig M. (2006). Addition of Pectin and Soy Soluble Polysaccharide Affects the Particle Size Distribution of Casein Suspensions Prepared from Acidified Skim Milk. J. Agric. Food Chem..

[11] Nakamura A., Furuta H., Kato M., Maeda H., Nagamatsu Y. (2003). Effect of Soybean Soluble Polysaccharides on the Stability of Milk Protein under Acidic Conditions. Food Hydrocoll..

[12] Wusigale, Liang L., Luo Y. (2020). Casein and Pectin: Structures, Interactions, and Applications. Trends Food Sci. Technol..

[13] Igartúa D.E., Cabezas D.M., Palazolo G.G. (2022). Effects of PH, Protein: Polysaccharide Ratio, and NaCl-Added Concentration on Whey Protein Isolate and Soluble Soybean Polysaccharides Electrostatic-Complexes Formation. Food Chem. Adv..

[14] Tang W., Liu C., Liu J., Hu L., Huang Y., Yuan L., Liu F., Pan S., Chen S., Bian S. (2020). Purification of Polysaccharide from Lentinus Edodes Water Extract by Membrane Separation and Its Chemical Composition and Structure Characterization. Food Hydrocoll..

[15] Atgié M., Garrigues J.C., Chennevière A., Masbernat O., Roger K. (2019). Gum Arabic in Solution: Composition and Multi-Scale Structures. Food Hydrocoll..

[16] Wang S., Zhao L., Li Q., Liu C., Han J., Zhu L., Zhu D., He Y., Liu H. (2019). Rheological Properties and Chain Conformation of Soy Hull Water-Soluble Polysaccharide Fractions Obtained by Gradient Alcohol Precipitation. Food Hydrocoll..

[17] Nakamura A., Yoshida R., Maeda H., Furuta H., Corredig M. (2004). Study of the Role of the Carbohydrate and Protein Moieties of Soy Soluble Polysaccharides in Their Emulsifying Properties. J. Agric. Food Chem..

[18] Ikeda S., Funami T., Zhang G. (2005). Visualizing Surface Active Hydrocolloids by Atomic Force Microscopy. Carbohydr. Polym..

[19] Wei Y., Cai Z., Wu M., Guo Y., Tao R., Li R., Wang P., Ma A., Zhang H. (2020). Comparative Studies on the Stabilization of Pea Protein Dispersions by Using Various Polysaccharides. Food Hydrocoll..

[20] Tian H., Zhao Q., He Z., Wang Z., Qin F., Zeng M., Chen J. (2021). Effects of Molecular Weight and Degree of Esterification of Soluble Soybean Polysaccharide on the Stability of Casein under Acidic Conditions. Foods.

[21] Liang W.-L., Liao J.S., Qi J.R., Jiang W.-X., Yang X.-Q. (2022). Physicochemical Characteristics and Functional Properties of High Methoxyl Pectin with Different Degree of Esterification. Food Chem..

[22] Dickinson E. (2006). Food Emulsions: Principles, Practices, and Techniques, Julian McClements, 2nd edition, CRC Press, Boca Raton, FL (2005), pp. 609, $149.95. Food Hydrocoll..

[23] Li J., Matsumoto S., Nakamura A., Maeda H., Matsumura Y. (2009). Characterization and Functional Properties of Sub-Fractions of Soluble Soybean Polysaccharides. Biosci. Biotechnol. Biochem..

[24] Wagoner T.B., Foegeding E.A. (2017). Whey Protein–Pectin Soluble Complexes for Beverage Applications. Food Hydrocoll..

[25] Zhao R.X., Qi J.R., Liu Q.R., Zeng W.Q., Yang X.Q. (2018). Fractionation and Characterization of Soluble Soybean Polysaccharide Esterified of Octenyl Succinic Anhydride and Its Effect as a Stabilizer in Acidified Milk Drinks. Food Hydrocoll..

[26] Nobuhara T., Matsumiya K., Nambu Y., Nakamura A., Fujii N., Matsumura Y. (2014). Stabilization of Milk Protein Dispersion by Soybean Soluble Polysaccharide under Acidic PH Conditions. Food Hydrocoll..

[27] Zhang M., Sun C., Li Q., Melton L., Shahidi F., Varelis P. (2019). Interaction Between the Polysaccharides and Proteins in Semisolid Food Systems. Encyclopedia of Food Chemistry.

[28] Nakamura A., Naeki R., Kikuchi M., Corredig M., Shima Y., Fujii N. (2023). Molecular Structures of High- and Low-Methoxy Water-Soluble Polysaccharides Derived from Peas and Their Functions for Stabilizing Milk Proteins under Acidic Conditions. Food Res. Int..

